# Perspectives of older adults with memory decline participating in a prolonged nightly fasting (PNF) pilot study: A qualitative exploration

**DOI:** 10.1017/cts.2025.63

**Published:** 2025-04-08

**Authors:** Dara L. James, Erica Ahlich, Molly Maxfield, Afton Kechter, Sarah E. James, Alexis M. Koskan, Dorothy D. Sears

**Affiliations:** 1 Arizona State University, Edson College of Nursing and Health Innovation, Phoenix, AZ, USA; 2 University of South Alabama, Department of Psychology, Mobile, AB, USA; 3 Eli Lilly and Company, Clinical Design, Indianapolis, IN, USA; 4 Mayo Clinic Arizona, Department of Radiation Oncology, Phoenix, AZ, USA; 5 Arizona State University, College of Health Solutions, Phoenix, AZ, USA

**Keywords:** Intermittent fasting, aging, cognition, sleep, clinical trial, qualitative research

## Abstract

**Introduction:**

Cognitive decline and sleep concerns are significant health issues among older adults. Nonpharmacological treatments to address these concerns are needed, particularly for older adults who are more likely to be prescribed multiple medications and experience adverse effects of additional drugs. The aim of the current qualitative study was to understand and document the experiences of older adults with subjective memory decline participating in prolonged nightly fasting (PNF) intervention.

**Methods:**

This single-group pilot study was conducted as a fully remote, 8-week, pre/postintervention. Postintervention, 16 participants (≥65 years) participated in semistructured qualitative exit interviews about their experiences with the PNF intervention. Interviews lasted approximately 20–30 minutes, were conducted by trained study staff, and then analyzed by the team to understand relevant themes.

**Results:**

Two major themes that emerged from the data were engagement with and perceived effects of the PNF intervention. Within these two themes, nine subthemes emerged: accountability; use of days off; feasibility; intervention tools; behavioral strategies; timing/routine; awareness; self-efficacy; and perceived health-related outcomes. Overall, interviews suggested strong engagement with the PNF intervention as well as a number of positive perceived effects of the intervention.

**Conclusions:**

These findings contribute to a broad field of intermittent fasting by exploring and understanding the direct experiences of older adults participating in PNF. Some participants identified challenges of participation, yet this qualitative approach can guide future PNF implementation with older adults. Notably, responses support the quantitative data suggesting that PNF is a feasible and acceptable intervention for older adults.

## Introduction

Although intermittent fasting (IF) for therapeutic purposes has been practiced for over a century [1], recent literature to advance understanding of outcomes associated with IF has grown tremendously. Alternative to making specific dietary changes, IF involves periods of hours and/or days with little to no caloric consumption [2–4], followed by a refeeding period. Thus, the focus of IF is on the *timing* of caloric consumption rather than the quality or quantity of dietary consumption [2]. There are various forms of IF (e.g., alternate day fasting [ADF], time-restricted eating [TRE], prolonged nightly fasting [PNF]), and accumulating evidence suggesting health benefits including improved glucose regulation, weight loss, reduced inflammation, improved sleep and cognition [2,4], and reduced cancer risk [5,6]. Most prior work examining the health outcome effects of IF has focused on quantitative measures including weight loss and metabolic outcomes [7] primarily among younger adults [8], largely overlooking older adults [2,9]. More work, particularly qualitative exploration, is needed to examine the experience and perceived benefits of IF among older adults.

Almost a quarter of the U.S. population is expected to be 65 or older by 2060 [10]. Older adulthood is associated with a greater risk of sleep disorders [11], which is further associated with morbidity and mortality, depression, and reduced quality of life [12,13]. Older age is also a risk factor for cognitive decline, impairment, and dementia [14,15]. Results of a longitudinal study of nearly 30,000 adults 50 years or older suggested that at approximately 70 years old, two-thirds of U.S. adults experience cognitive impairment [16]. Given the substantial need in this population and the empirical support for IF targeting these concerns among young adults, research examining the extent to which the benefits of IF generalize to the older adult population is needed.

While researchers are investigating the mechanistic pathways driving the benefits of IF, recent literature promotes TRE, fasting with circadian rhythm alignment, when all calories are consumed within a specific window of time. Avoiding the consumption of calories during the evenings and late at night (outside of typical circadian rhythms) has been linked to reduced risk for cancer and cardiometabolic disease [17–19]. Therefore, PNF, a type of TRE in which individuals limit caloric consumption to daytime hours, avoiding caloric consumption at night, aligns naturally with individuals’ circadian rhythms [20–22]. PNF can potentially improve sleep [20] and may also assist in diminishing sleep- and cognition-related disorders [23,24]. Primary quantitative outcomes of the parent study found that 8 weeks of PNF improved cognitive function (*p* = 0.02) and reduced insomnia (*p* = 0.04) among older adults with subjective memory decline (N = 18; age ≥ 65 yrs) [21].

In sum, evidence to date supports PNF to improve markers of physical and cognitive functioning. Here, in our qualitative exploration based on in-depth interviews, we extend this line of inquiry to better understand participant experiences of engagement (e.g., barriers, facilitators) and perceived effects (e.g., health outcomes) with the 8-week PNF protocol. While data from the current parent study found that, on average, adherence to PNF was strong; there was variability across participants, indicating potential for improvement (e.g., ranging from 70 to 100% adherence [21]). Similarly, a recent scoping review reported superior adherence to IF protocols (∼23–24%) compared to caloric restriction protocols (∼5–21%) among adults [2], yet adherence rates could be improved with a better understanding of how indvidual participants expereinced the protocol and the potential barriers faced. Qualitative approaches are uniquely suited for this further line of inquiry [25], as such interviews provide opportunities to gain a more nuanced understanding of experiences and engagement with the intervention. Qualitative methods are especially important when adapting interventions to new target populations or settings [26] and are consistent with a person-based approach to assessing and enhancing the acceptability and feasibility of novel interventions [27]. Thus, the current qualitative study aimed to explore the uptake, acceptance, perceived effects, and facilitators and barriers of the PNF intervention and, further, to help guide the refinement of future PNF interventions for scalability and a wide participant audience.

## Materials and methods

**Figure 1.**
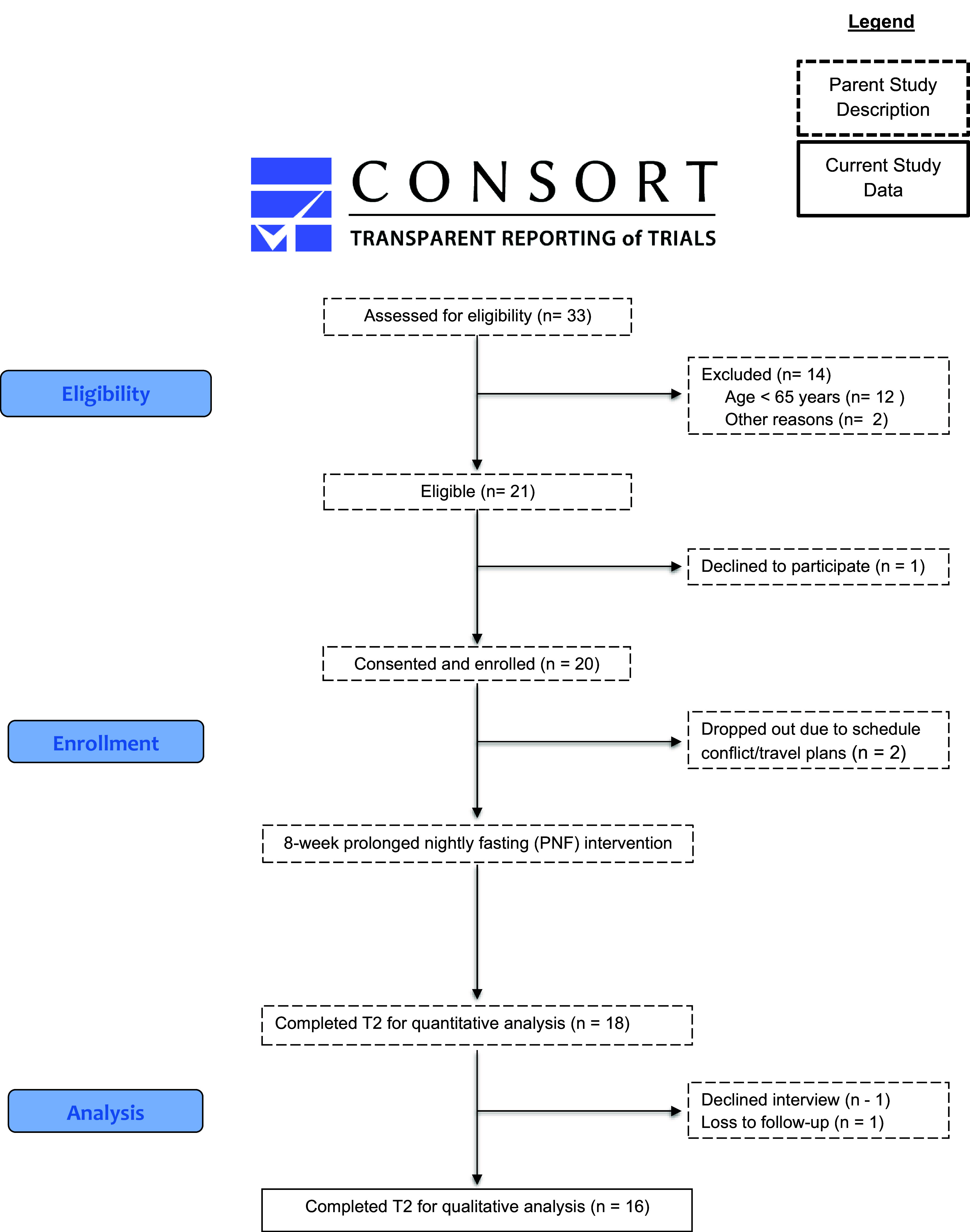
Participant flow chart.

The parent 8-week PNF pilot study was conducted as a remotely delivered, single-group, pre/postintervention (see Fig. 1). Quantitative primary and secondary study results are reported elsewhere [21]. The current qualitative analysis applied standard reporting guidelines [28] (see supplemental materials Table 1. Consolidated Criteria for Reporting Qualitative Studies; COREQ). The study was approved by the Institutional Review Board at Arizona State University (Protocol #STUDY00012888) and was registered on Clinicaltrials.gov (NCT04938778).

### Participants

Intervention details for the parent study about participant recruitment and a full list of inclusion and exclusion criteria can be found in another manuscript [21]. Primary inclusion criteria were: 1) self-reported “memory not as good as it used to be;” 2) ≥65 years old; and 3) owned a smartphone with access to WiFi and Zoom; primary exclusion criteria were: 1) neurocognitive disorder; 2) already routinely fasting for 14+ hours a night; 3) diabetes, eating disorder, contradicting medical condition. All eligible participants were emailed an informed consent document to sign and return.

### Intervention

Participants in the parent study were asked to engage in an 8-week PNF intervention in which they fasted 6 days per week (with one day off per week) for 14 hours per night, followed by a 10-hour eating window for 6 days each week. They were asked to start their fast no later than 8:00 p.m.; but could otherwise be flexible with their fasting start/stop times. During the fast, participants could drink water, coffee, or tea (without milk products or sweeteners/artificial sweeteners). Participants were asked to consume food/beverages as they normally would during their eating window (i.e., no changes to diet quantity or quality).

**Figure 2.**
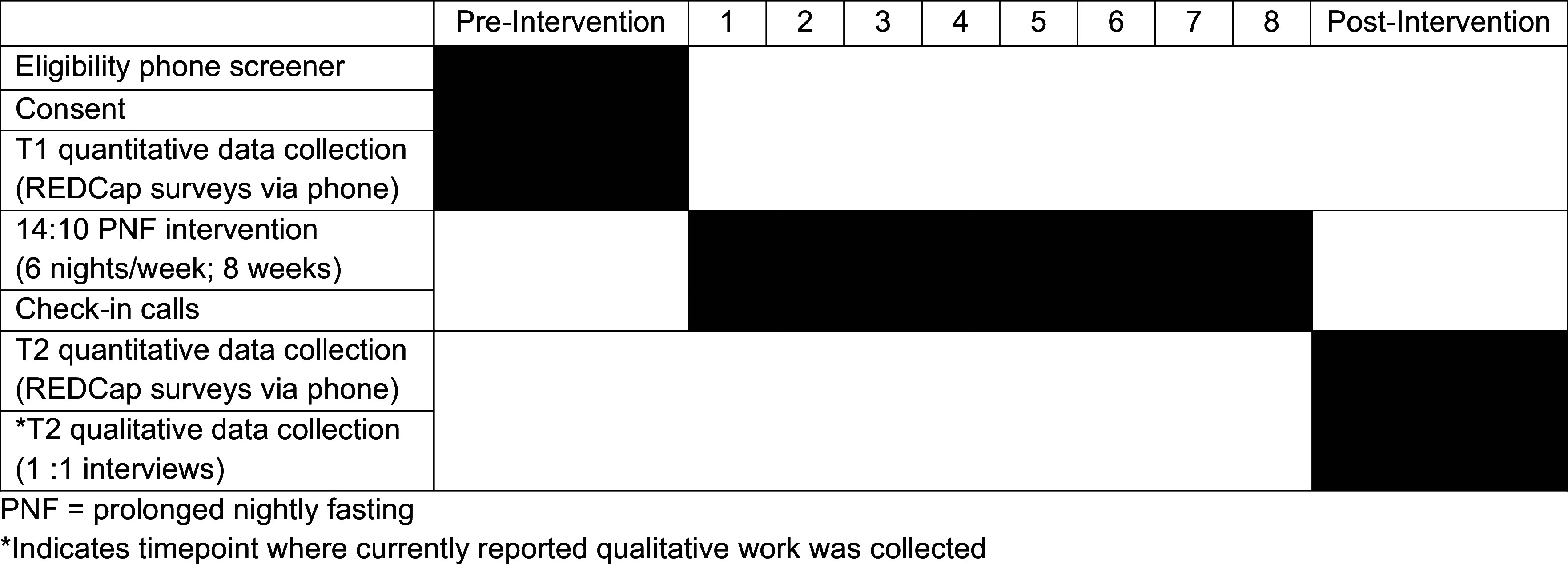
Intervention components and timeline by week.

Participants were mailed an information packet prior to the study start including a weekly and monthly calendar to track fasting dates and start/stop times. Study staff conducted weekly check-in phone calls (approximately 10 minutes) in which participants discussed their fasting times; some participants elected to report this information via text (see Fig. 2. Intervention Components and Timeline by Week).

### Postintervention interviews

Post-intervention, all participants in the parent study were invited to complete a semistructured qualitative exit interview (see supplemental materials Table 2. Interview Guide). One study team member (DLJ) with doctoral-level qualitative training conducted and audio recorded the study interviews using Zoom. This team member did not have a relationship with the participants prior to study commencement nor did the participants have information about the interviewer, their characteristics, or the study goals prior to the interviews being conducted. No one else was present during the interviews. One-time interviews lasted (i.e., no repeat interviews), on average, between 20 and 30 minutes. Interview questions (10 in total) were developed by the primary investigator and senior author team, who previously used similar questions for another PNF pilot study among older postmenopausal women. For transcription purposes, interviews were sent to a professional transcription company; additional field notes were not made during the interviews, nor were interviews returned to the participants.

### Analysis

Four study team members conducted a qualitative content analysis of interview data [29]. We took a team-based approach to identify codes in the qualitative data. The team reviewed two transcripts together in a group meeting and discussed preliminary themes and codes using a mix of both deductive (based on the interview guide) and inductive (as other themes emerged) analytic practices to identify themes and subthemes [30]. Once the group reached a consensus on the coding strategy, the first author assigned a set of interviews to four research team members who reviewed and coded the interviews. To minimize bias, they each reviewed interviews independently, without access to one another’s files [31]. This was followed by a series of group consensus meetings by all four coders, to discuss how they coded the interviews. When they identified coding discrepancies, they resolved differences and updated the coding guide. As such, the coding scheme evolved with each application to a new interview. When no new themes emerged (i.e., saturation), the remaining interviews were split into teams of two coders each. All coders were female, Ph.D.-level experts and had prior qualitative coding experience in their respective fields. During group meetings, coders discussed any potential personal biases that might have influenced codes. Two coders summarized the coded materials and identified quotes representing emerging themes.

## Results

### Demographics

Of the 20 participants who enrolled in the parent study, 18 completed the eight-week PNF intervention (90% completion rate), and 16 completed the semistructured postintervention interviews. Of those who did not complete the postintervention interview, one declined, and one was lost to follow-up (see Fig. 1. Participant Flow Chart). Of those who completed the interviews, participant ages ranged from 65 to 84 years old with a mean age of 70.2 years old. The baseline mean BMI was 30 kg/m^2^. Participant interviewees were non-Hispanic White (100%), mostly female (94%), married (44%), unemployed (38%), lived independently without a caretaker (88%) and had an income ≥$100,000 (25%). Additional details of the full sample are reported elsewhere [20].

### Qualitative analysis

The current qualitative study yielded two primary themes with nine subthemes. The primary themes were 1) engagement with the intervention and 2) perceived effects of the intervention. Each theme and additional subthemes are discussed below with accompanying exemplar quotes from participants.

### Theme 1: engagement with the intervention

Participants described ways in which they engaged with the PNF intervention. Within this theme, six additional subthemes provided a more comprehensive understanding of their engagement which includes the following: 1) accountability; 2) feasibility; 3) use of the day off; 4) intervention tools; 5) behavioral strategies; 6) timing/routine.

#### Accountability

Participants described their feelings of connection to the program and accountability, largely due to their experiences speaking with study staff during the weekly check-in calls:

“She [study staff] checked in with me—we had a little chat once a week, and I think that was appropriate. Probably that, and the reporting certainly introduced a level of accountability, and absolutely I need that. I need someone over me.” [P8-F-73]

“I would say that kind of calling, whether it’s really for the information or just to keep you connected, really works to keep you in the program.” [P12-F-67]

“The fact that I like to make people happy…so I wanted to make sure if she [staff] did either text or call or email that I was on track, so I could say, “Yes. I”m doing exactly what I need to do.’ There was that accountability.” [P11-F-70]

“If I know somebody’s gonna call me, I wanna have a good report. I don’t wanna say, “Oh. I really messed up.” I don’t like to report failure, so just the fact that my health coach was gonna call once a week, and [staff name] was gonna call once a week, kept me on the program.” [P17-F-70]

Others commented on the benefits of weekly check-in during the early stages of habit formation.

“I think it [weekly check-ins] helped. Yes. Because it was that accountability, especially until you make something into a real habit.” [P16-F-69]

“Right. I believe that it did [check-ins helped] because my motivation for doing it was the study, but I don’t believe that I needed it [check ins] once I decided that this [study participation] is going to be my own personal commitment to it.” [P15-F-66]

#### Feasibility

Participants described the feasibility of integrating PNF into their daily lives. Participants unanimously provided positive responses when asked about any overall difficulties engaging in PNF, describing their ease in completing the intervention.

“Really, there wasn’t anything hard about it at all…Just follow directions and instructions.” [P6-F-70]

“…it was easy ’cause it was like a cutoff time…the next day, I would remember to not eat until those two hours had elapsed after the—If it was 6:00, then it would be 8:00, and if it was 7:00, it’d be 9:00. It’s easy to keep track of…” [P17-F-70]

When asked how to describe the level of difficulty in PNF, another participant noted,

“…in general, it’s kind of what I like, and it seemed comfortable to me. I’m gonna try to keep it up regardless of whether I continue in a study or not, or keep it up in a reasonable way.” [P19-F-75]

Another noted,

“Honestly, there was nothing that was all that hard. I mean, there were a couple of days where I was hungry earlier and wanted to eat, but it wasn’t like it was hard. It was just, “Oh, I cannot do that [eat] yet.” I do not think there was anything that was actually hard.” [P16-F-69]

One participant described the PNF protocol as being easier to adhere to compared to other programs they had participated in.

“…over the years, I’ve participated in several …directed programs…I think what I like about this, it was very well-thought-out and easy to follow…I think that it’s something that I would look forward to making part of the rest of my life.” [P11-F-70]

#### Use of the day off

Some participants shared how having one day off per week enhanced their engagement with the PNF intervention. When asked how often they used the days off that were allowed as part of the protocol, participants responded:

“We didn’t [use our days off] very often. For two days, we used cheat day when we had events, like a bar mitzvah party, and we ate later. When we fasted on our Jewish holiday, then we broke the fast later. Not very often.” [P3-M-78]

Another participant noted,

“I rarely took a day off. There was one day where she [a staff member] said, “You can have one day off.” I mean, I think the only other time I did that [break the fast] was by accident. I only went over maybe 20 minutes to a half hour, not even long.” [P16-F-69]

#### Intervention tools

Participants described how they used the fasting and eating tracking sheets (one of which allowed them to record daily eating start and stop times; one of which was the entire 8 weeks where they tracked their once weekly day off). Using these forms enhanced their adherence to the PNF protocol.

“The one [tracking form] that had every day… where you would put your day off… I marked it, probably, at the end of the week or every three or four days…And then on the other days, I’d put X’s that I did them [fasted]. I did use that as a visual counter.” [P12-F-67]

Another described using one of the calendars provided by the study.

“I used the calendar. I printed it off and made a copy for the entire eight weeks…I carry a bag with me to work every day, and I had it in my bag. I’d pull it out when I started eating and just mark the time and then put it back. …Then do the same thing at the end of the night when I finished eating. I’d mark the time.” [P16-F-69]

While some participants found the various fasting tracking tools to be helpful, others did not find them valuable or were indifferent:

“I don’t think any of them were that helpful… all I needed was just a small piece of paper to keep track of what hours I did eat, and they were basically the same every day.” [P2-F-67]

“The paper where you record when you stopped eating and started eating was a little confusing or maybe not efficient.” [P8-F-73]

“I ended up just tossing it all. Once the first phone call happened I found out, “Oh you’re just gonna get the information from me.” I texted my little notes a week at a time” [P5-F-68]

#### Behavioral strategies

During the interview, many participants described cognitive and behavioral strategies that they used to enhance their adherence to the PNF intervention. Several described drinking water to increase their satiety:

“I can’t think of any real difficulties that I did have with it. If I had a little hunger pang after I had quit eating, I would just drink water.” [P2-F-67]

“I’m not a big snacker at night, but if I did get hungry, I was able to let it pass. I would just drink some water.” [P10-F-65]

Others described substituting other beverages, such as tea, to increase their adherence to the protocol.

“I usually have a glass of wine. I didn’t do that. I switched over to hot tea, which was helpful, but it wasn’t the relaxing wine that I’m used to having. That was the most difficult part. But I got used to it. By week two, I was okay with that.” [P9-F-73]

“Not having coffee first…With cream…I cannot drink it without. Instead I would start with green tea ’cause I could tolerate that without anything in it….” [P20-F-65]

To cope with hunger cues late at night, when participants had previously been snacking, one participant described a strategy of going to bed earlier.

“If I’d get hungry at 10:00, I had to restrain myself and not have a snack. I usually would just try to go to sleep— so that I’d be unconscious and not hungry.” [P17-F-70]

Another described using a personal mantra to boost motivation.

“I could feel a little tussle going on within me [wanting to break the fast]. But then I would tell myself, “You can do this. You can do this.” That would go on for maybe—not very long, 30 s. Then I’d get past that, and it was a triumph.” [P8-F-73]

To easily remember the beginning or end of a fasting period, participants identified using a phone alarm or electronic notes:

“That was easy enough to keep track of anyhow. I just set the alarm on my phone.” [P8-F-73]

“Because I’m an early-morning, late-night person, and I just get involved in my day, so I would have to set an alarm. I think that’s what I would have to do, is just set a little alarm and say, “Okay. it’s time for you to have some fruit or cereal or something to start the day.” [P11-F-70]

Finally, one participant described modifying the home environment to facilitate adherence:

“Being used to—if at 8:30 or 9 o’clock at night—because I am a late person—if I get an urge, I would get—I love celery and peanut butter and so I had to make sure that the peanut butter jar was tight, so I couldn’t get into it.” [P11-F-70]

#### Timing/Routine

An important subtheme emerged from participant comments that described barriers and facilitators to intervention adherence based on schedules, particularly as it related to the timing of meals and how they modified their former routines. Many participants described a particular PNF challenge and how they identified solutions by schedule adjustment. Participants described how, at times, it was difficult to adhere to the PNF schedule; a few noted the difficulty of fasting in the mornings.

“Waiting until 10:00 in the morning to eat wasn’t hard except that I often was in the middle of work and I would have meetings that start at 10:00, so that interfered a little bit with my work time, but then I found ways around that” [P15-F-66]

When breakfast was consumed later than what they had become accustomed to, particularly during mornings, they shifted to other regular mealtimes.

“The hardest thing I found was because I was eating breakfast at 10:00 a.m. I have this habit of eating breakfast in the morning earlier, then lunch around noon. I had a hard time figuring out when to eat lunch because my habit of eating lunch at noon was like, well, I just ate two hours ago, and I don’t need to eat right now…Then I would eat again a little bit later in the afternoon. I should’ve just moved lunch back, but I never really got into a routine with it. So that was my hardest part, figuring out when to eat lunch.” [P15-F-66]

“At first I think I was starting eating later in the morning like 10 and then stopping at eight. Then it’s after the next couple of weeks, it evolved where I was stopping eating at five or six in the evening. Then I could start earlier in the morning.” [P20-F-65]

Participants described their challenges related to habits specific to coffee:

“The hardest part for me was not being able to get up and have coffee [with cream or sugar]. Because I would try to—I would look and see when I stopped eating and figure out my 14 hours for when I could have coffee.” [P7-F-84]

“I didn’t find the 14 hours [of fasting] to be all that difficult, except for the milk and coffee. That was a little bit hard. I still can’t say I like black coffee. I found I was eating earlier because I wanted to be able to have my coffee in the morning.” [P10-F-65]

Other participants described the difficulty of fasting at night:

“The fasting itself wasn’t an issue. It was more the issue of the 8:00 [p.m.] cut-off because, sometimes, if we were invited out to eat, it’s difficult to have that 8:00. The actual fasting for 14 hours is not difficult. I’m in a lot of sports, so sometimes, if I knew I had to cut off early to get up to decide whether to do my morning shake or early or after I go to do my sports was a little tough.” [P3-M-78]

“I have a lot of family get-togethers and parties and meetings and things where eating takes place after 6:00. I would feel like that was kind of limited. I could adjust it on the other end, but I’m just saying it’s somewhat difficult. I could do it. I would maybe just eat before those occasions, but then eating is a social act at times.” [P17-F-70]

At times, the prescribed eating window differed from the timing of hunger cues, and some participants described the anticipation of this difference as a challenge:

“Sometimes I wanted to have something late, or I just wanted to eat breakfast early, because I was really hungry. I didn’t, because I knew that I would have to eat dinner earlier, and then I would be really hungry at night.” [P9-F-73]

“I literally wake up hungry and the idea of trying to postpone that until 8:00 so I’m not having to finish eating at 4:00 in the afternoon or whatever, I could wake up and have my kombucha tea at 7:00, and by 7:15 I’m eating something. That’s more normal for me.” [P5-F-68]

Once the initial adjustment period ended, one participant described preferring this new routine.

“I started making sure that I started getting something in my stomach at least by 8 o’clock, so I was finished by 6:00…I was just talking to a friend the other day. I really enjoyed that, so I really need to be back on that track.” [P11-F-70]

### Theme 2: Perceived effects of the PNF intervention

The other major theme of the interviews was the perceived effects of the PNF intervention. Within this broader theme, three subthemes emerged: 1) awareness; 2) self-efficacy; and 3) perceived health-related outcomes.

#### Awareness

Over half of the participants expressed noticing a shift in awareness, specifically with regard to hunger cues, differentiating between different bodily cues, and time as it related to eating. Several participants noted awareness changes related to experiences of hunger.

“I think that it really does depend on how aware you are of things. I am usually never hungry in the morning. When I am hungry in the morning, I became very aware of that [my hunger].” [P1-F-65]

“Well, it taught me the difference between an acid stomach and hunger. I have GERD, so sometimes it’s difficult for me to know when it’s just GERD or if I’m really hungry. When I got up in the mornings, I knew I was hungry. It’s more clear now.” [P9-F-73]

“I thought about how I didn’t need to eat while I was watching TV as much. That it was fine not to [eat], and that I didn’t really get hungry. But it [drive to eat] was just more out of boredom.” [P3-M-78]

Another described experiencing less preoccupation with food throughout the day:

“Primarily, I didn’t think about food as much. How you go through your day thinking, ooh, what can I eat? I just didn’t think about it as much.” [P16-F-69]

Others noted changes in their awareness of the time as it related to eating. Remembering the PNF intervention made them think about the timing of meals so that they could adhere to the study.

“I think the one thing I noticed, especially when I would go out for dinner, I became very aware of time. I’d be out at a restaurant and I’d be asking my friends, “What time is it?” so I know when I stopped eating what time I’d have to start the next day.” [P10-F-65]

“I do not normally eat past 8:00 or 8:30 anyway. If I’m going to snack, I try to do it before 8:00, so it [the intervention] made me more mindful of the clock. If I slipped past the time I wanted to stop eating, I just didn’t snack on anything that night…It made me more aware of the time to stop eating so that I’d know when to start eating the next day.” [P7-F-84]

“I think, it [the intervention] just made me stop and think about what time of the day it was …what I learned was I need to pay better attention, and so I’ve started writing things down, what I eat, when I eat, and things like that, and making sure I eat five to six little times a day, but watching the time, and I think that’s made the difference.” [P11-F-70]

### Self-efficacy

The second subtheme was related to self-efficacy. One participant described experiencing a stronger sense of control over eating after participating in the PNF study:

“Growing up, I’ve always had problems with overeating. Recent years, it’s a little better, but I think I’ve felt a little bit better for myself seeing that I had sort of a routine, and good, healthy foods to eat at specific times. And I wasn’t nibbling all day. That made me feel better about myself…Control is a silly word, but I feel like it’s a little bit more manageable, and I feel good psychologically about that, that I’m just not eating from the time I get up until the time I go to bed…” [P19-F-75]

One participant commented that changes made each day facilitated changes on the following day:

“Each day that I got through and maintained the fast, I felt good about my eating habits.” [P8-F-73]

The same participant noticed a reduction in craving-induced eating and other unplanned eating:

“I live alone. I tend to feed myself whenever the urge hits me or even when it doesn’t. Having a cutoff time kept me from eating a lot of bad stuff, the pick-up-quick stuff that quite often isn’t so good for you.” [P8-F-73]

### Perceived health-related outcomes

The final subtheme represents important participant observations related to psychological, behavioral, or physical changes because of the PNF intervention. Several participants commented on noticing changes in cognitive functioning:

“The fact that my mind felt so clear… I do not think it was—what’s that effect when you—the Placebo effect—it could not happen the first week. It was definitely about three weeks in, and then I was like, “Wow. Wow. This is really cool. This feels really good.” [P12-F-67]

“I felt I was much clearer, sharper. I felt I didn’t have as much lag time. Sometimes I can feel myself using a number of switches in my brain, you know, when you try to find that word that you always want but you cannot find it. I just really felt “on,” and, actually, that would be an impetus for me staying on it for six months. I felt it would really help my brain, more than anything else…” [P12-F-67]

“I will tell you this, I was very worried about my memory before the program. I had left things in the oven twice and gone away in my car and had to have a neighbor come and turn my oven off once…I didn’t do anything like that during the eight weeks, so well, that’s an improvement.” [P17-F-70]

Participants perceived changes in their digestion. For example, one participant noted:

“I think my system and plumbing, internal plumbing all worked better when I was on that schedule, which is probably why I’m sticking with it.” [P16-F-69]

The same participant noted changes in sleep due to study participation:

“I feel like I’ve fallen asleep a little faster and stayed asleep a little better. There was more time between the time I finished eating and the time I went to bed….That’s what I thought because I never ended up never going to bed on a full stomach.” [P16-F-69]

## Discussion

While the body of literature focused on IF and PNF is rapidly increasing, the vast majority of results focus on quantitative results [6]. Our research uniquely explored participants’ experiences through a qualitative lens; study results provide an in-depth, firsthand exploration of participation in a PNF intervention among older adults with subjective memory decline shedding light on subjective responses and health outcomes. As the first known PNF qualitative research, this study provides a unique and nuanced perspective on participants’ lived experiences, going beyond quantitative metrics to capture the complexities and successes of engaging in PNF as a daily regimen.

This research delves into participants’ motivations, challenges, and adaptations throughout the PNF intervention. The findings highlight shifts in habits, self-perception, and attitudes toward engaging in the PNF protocol. Additionally, this work addresses how participants navigated barriers, such as personal scheduling constraints, that influenced their adherence to and experiences of PNF. The insights gained contribute to a more comprehensive understanding of PNF as a behavioral lifestyle intervention. By capturing these qualitative dimensions, this research fills a crucial gap, providing actionable information to inform future research.

In line with recent qualitative work exploring TRE, our work demonstrates similar themes of participant experiences postfasting, including categories of intervention preparation, adaptation, challenges, and maintenance [32]. In addition, a second qualitative study of participants who had engaged in at least 3 months of TRE, researchers identified the following themes: 1) dissatisfaction and resistance to prior or traditional dietary approaches; 2) perceived broader health benefits of time-restricted eating (TRE); 3) principles of TRE deemed logical; 4) low to no cost of adoption; 5) manageable psychosocial barriers; 6) being nonrestrictive and easy to use; and 7) compatibility with personal lifestyle [33]. Although the body of qualitative work with respect to IF (e.g., PNF, TRE) is just developing, here we note that it is of interest that these early studies found similar themes across participant experiences. Further inquiry and collaboration are needed to continue to drive this important line of work.

Study findings highlight the relative ease with which PNF was implemented into daily life. This finding corresponds with recent data from populations with potential barriers to TRE such as cancer-related fatigue, wherein participants also expressed that a similar TRE regimen was considered feasible, well-received and, further, that participants were willing to continue [34]. In the current study, many participants commented on the protocol’s simplicity and need for minimal recording and their ability to readily identify strategies to adapt for intervention adherence and sustainability. Notable strategies included beverage substitution (e.g., water, tea), use of motivating mantras, alarms to signify the end or beginning of the fast, and adjusting the eating window to best suit personal schedules and preferences. Future PNF studies should examine the efficacy of using these strategies to increase fasting adherence. This kind of iterative refinement of behavior intervention protocols is consistent with a person-based approach that incorporates the experiences [27], attitudes, and needs of the participant population that will hopefully use and benefit from PNF.

Additionally, participants described tools and activities that enhanced intervention adherence. For example, weekly check-ins enhanced emotional and social support to engage with the intervention, giving them a sense of connection with and accountability to the study. Past research has described that such support benefits participants by making them feel less alone with their fasting experiences [35]. Additionally, having one “day off” per week may have enhanced participants’ study adherence. A recent study cited three clinical trials that offer one “cheat day” per week, as this may enhance study protocol adherence [36]. The intervention tools were available to help individuals self-monitor meal timing. Past research shows that tools to enhance self-monitoring, such as phone apps, help individuals adhere to fasting; on the other hand, removing the use of such apps led to participant disengagement [35]. Future IF and PNF interventions should consider implementing such easy-to-employ tools and check-ins to help participants remain engaged. Additionally, researchers may want to provide further guidance on navigating social interactions while fasting, as this was noted as a barrier to fasting adherence in previous research [35].

This perceived physical benefits of PNF also align with recent research suggesting TRE may have neuroprotective effects on improved cognition [21], improved sleep [20,21] reduced neuroinflammatory markers associated with dementia [37], and improved GERD symptoms of heartburn and regurgitation [38]. As a rapidly growing area, exploring the potential indirect effects of IF, specifically, PNF on other, and more long-term, physical and mental health outcomes will be important.

### Limitations

The current pilot study was limited by the small sample size and relatively homogeneous population. However, the study team reached information saturation within this small sample. Future research should engage a more diverse sample of older adults in PNF research to enhance our understanding of participant responses to this approach and the generalizability of findings. Additionally, the study duration of 8 weeks may have limited a more in-depth understanding of the participants’ experience; future research should consider exploring a longer PNF intervention.

## Conclusion

This body of qualitative research work adds a unique approach and critical understanding to the field of PNF, particularly in how it relates to the experiences of older adults. It offers nuanced insights into the personal, psychological, and social factors that influence engagement, adherence, perception, and outcomes to the PNF intervention. By focusing on lived experiences, this work highlights the complex interplay between individual motivations, challenges, and the broader context in which IF, particularly PNF, is practiced. Together with the quantitative findings [21], this remotely delivered, low-cost, low-burden intervention appears to be both feasible and associated with a number of positive health-related outcomes among older adults with memory decline. Future research should explore strategies to optimize implementation and sustainability, ensuring that the benefits of PNF can be effectively realized across diverse populations and settings.

## Supporting information

James et al. supplementary material James et al. supplementary material

## Supplementary material

The supplementary material for this article can be found at https://doi.org/10.1017/cts.2025.63
