# Supplementary material for: Perspectives of older adults with memory decline participating in a prolonged nightly fasting (PNF) pilot study: A qualitative exploration
Source: J Clin Transl Sci. 2025 Apr 8;9(1):e112. doi: 10.1017/cts.2025.63 (PMC12171933; doi:10.1017/cts.2025.63)
Supplement: James et al. supplementary material [file S2059866125000639sup001.docx]

**Supplemental Materials**

S1. Consolidated Criteria for Reporting Qualitative Studies (COREQ)

| No.  Item | Guide questions/description | Page Reported |
| --- | --- | --- |
| Domain 1: Research team and reﬂexivity | |  |
| *Personal Characteristics* | |  |
| 1. Interviewer/ facilitator | Which author/s conducted the interview or focus group? | p. 6 |
| 2. Credentials | What were the researcher’s credentials? E.g. PhD, MD | p. 6 |
| 3. Occupation | What was their occupation at the time of the study? | p. 6 |
| 4. Gender | Was the researcher male or female? | p. 6 |
| 5. Experience and training | What experience or training did the researcher have? | p. 6 |
| *Relationship with participants* | |  |
| 6. Relationship established | Was a relationship established prior to study commencement? | p. 6 |
| 7. Participant knowledge of the interviewer | What did the participants know about the researcher? e.g. personal goals, reasons for doing the research | p. 6 |
| 8. Interviewer characteristics | What characteristics were reported about the interviewer/facilitator? e.g. Bias, assumptions, reasons and interests in the research topic | p. 6 |
| Domain 2: study design | |  |
| *Theoretical framework* | |  |
| 9. Methodological orientation and Theory | What methodological orientation was stated to underpin the study? e.g. grounded theory, discourse analysis, ethnography, phenomenology, content analysis | p. 7 |
| *Participant selection* | |  |
| 10. Sampling | How were participants selected? e.g. purposive, convenience, consecutive, snowball | p. 5 |
| 11. Method of approach | How were participants approached? e.g. face-to-face, telephone, mail, email | p. 5 |
| 12. Sample size | How many participants were in the study? | p. 8 |
| 13. Non-participation | How many people refused to participate or dropped out? Reasons? | p. 8 |
| *Setting* | |  |
| 14. Setting of data collection | Where was the data collected? e.g. home, clinic, workplace | p. 6 |
| 15. Presence of non-participants | Was anyone else present besides the participants and researchers? | p. 6 |
| 16. Description of sample | What are the important characteristics of the sample? e.g. demographic data, date | p. 8 |
| *Data collection* | |  |
| 17. Interview guide | Were questions, prompts, guides provided by the authors? Was it pilot tested? | p. 6-7; supplemental materials |
| 18. Repeat interviews | Were repeat interviews carried out? If yes, how many? | p. 6 |
| 19. Audio/visual recording | Did the research use audio or visual recording to collect the data? | p. 6 |
| 20. Field notes | Were ﬁeld notes made during and/or after the interview or focus group? | p. 7 |
| 21. Duration | What was the duration of the interviews or focus group? | p. 6 |
| 22. Data saturation | Was data saturation discussed? | p. 7 |
| 23. Transcripts returned | Were transcripts returned to participants for comment and/or correction? | p. 7 |
| Domain 3: analysis and ﬁndings | |  |
| *Data analysis* | |  |
| 24. Number of data coders | How many data coders coded the data? | p. 7 |
| 25. Description of the coding tree | Did authors provide a description of the coding tree? | p. 7-22 |
| 26. Derivation of themes | Were themes identiﬁed in advance or derived from the data? | p. 8 |
| 27. Software | What software, if applicable, was used to manage the data? | NA |
| 28. Participant checking | Did participants provide feedback on the ﬁndings? | NA |
| *Reporting* |  |  |
| 29. Quotations presented | Were participant quotations presented to illustrate the themes/ﬁndings? Was each quotation identiﬁed? e.g. participant number | p. 8-22 |
| 30. Data and ﬁndings consistent | Was there consistency between the data presented and the ﬁndings? | p. 22-26 |
| 31. Clarity of major themes | Were major themes clearly presented in the ﬁndings? | p. 7-22 |
| 32. Clarity of minor themes | Is there a description of diverse cases or discussion of minor themes? | p. 7-22 |

S2: Interview Guide

1. Do you think fasting 14 hours helped improve your eating behaviors?

A. Not at all

B. Somewhat neutral

C. Much

D. Very much

Please explain your response.

2. How difficult would it be for you to continue with this 14 hour fasting schedule for a full 6 months?

A. Very Easy

B. Easy

C. Somewhat Difficult

D. Very Difficult

Please explain your response.

3. Do you feel the coaching calls helped you stick to the 14 hours fasting call?

A. Not at all

B. Somewhat

C. Neutral

D. Yes

E. Yes, very much

What was helpful/not helpful about the coaching calls?

4. How many coaching calls would you prefer to receive each week?

A. 1 call

B. 2 calls

C. 3 calls

D. 4 calls

E. 5 calls

5. What type of tool from the participant packet was most helpful?

A. Clock

B. Weekly Calendar

C. Monthly Calendar

D. Eating Window

Why was it most helpful?

6. What was the hardest thing about following the 14 hour nightly fast?

7. Did you notice any changes in your thoughts or behaviors?

A. Not at all

B. Somewhat

C. Neutral

D. Yes

E. Yes, very much

Please explain any changes you had in your thoughts and behavior.

8. How would you rate your experience with the 14 hour nightly fast?

A. Very pleasant

B. Somewhat pleasant

C. Neither

D. Somewhat unpleasant

E. Very unpleasant

9. Is there anything else that you feel is important for us to know about your experience with the Think FAST study?
